# Identification and characterisation of putative seminal fluid proteins from male reproductive tissue EST libraries in tiger beetles

**DOI:** 10.1186/s12864-015-1619-9

**Published:** 2015-05-16

**Authors:** María Juliana Rodríguez-García, Vilmar Machado, José Galián

**Affiliations:** Department of Zoology and Physical Anthropology, Faculty of Veterinary, University of Murcia, Campus Mare Nostrum, E-30100 Murcia, Spain

**Keywords:** Seminal fluid proteins, Adephagan, Tiger beetles, Expressed sequences tags, Gene expression

## Abstract

**Background:**

The study of proteins transferred through semen can provide important information for biological questions such as adaptive evolution, the origin of new species and species richness. The objective of this study was to identify seminal fluid proteins (SFPs) that may contribute to the study of the reproductive system of tiger beetles (cicindelids), a group of more than 2,500 species distributed worldwide that occupy a great diversity of habitats.

**Results:**

Two cDNA libraries were constructed from the male gonads of *Calomera littoralis* and *Cephalota litorea.* Expressed sequence tags (ESTs) were analysed by bioinformatics approaches and 14 unigenes were selected as candidate SFPs, which were submitted to Reverse Transcription Polymerase Chain Reaction (RT-PCR) to identify patterns of tissue-specific expression. We have identified four novel putative SFPs of cicindelids, of which similarity searches did not show homologues with known function. However, two of the protein classes (immune response and hormone) predicted by Protfun are similar to SFPs reported in other insects. Searches for homology in other cicindelids showed one lineage specific SFPs (rapidly evolving proteins), only present in the closely related species *C. littoralis* and *Lophyra flexuosa* and two conserved SFP present in other tiger beetles species tested.

**Conclusions:**

This work represents the first characterisation of putative SFPs in Adephagan species of the order Coleoptera. The results will serve as a foundation for further studies aimed to understand gene (and protein) functions and their evolutionary implications in this group of ecologically relevant beetles.

## Background

Tiger beetles or cicindelids belong to the Adephaga, a suborder of Coleoptera that includes conspicuous, brightly coloured, non-pest species that are significant components of ecosystems, being important links in food chains. Cicindelids are organisms that are commonly used as bioindicators, as their presence or absence can provide information on the quality, alterations and successional stage of habitats, and are considered to be good bioindicators of general biodiversity [1–5]. There have been more than 2,500 species of cicindelid beetles described [6], which are distributed worldwide (except for Tasmania, Antarctica, and some Oceanic islands) and occupy a great diversity of habitats (alpine meadows, dessert grassland, among others) [7, 8]. Nevertheless, they do not show a homogenous pattern of distribution with relation to latitude or biogeography [6] and species tend to specialise in particular habitats [1]. They have several mechanisms or adaptations to reduce competition and contribute to maintain species richness; seasonal temporal segregation (life cycles), spatial segregation (differences in habitat or microhabitats preferences) [9] and temporal partitioning on the diel scale (diel activity patterns) have been previously studied as the most common strategies.

Tiger beetles have been deeply studied at different levels: (i) morphology [10], (ii) taxonomy [11–13], (iii) biology [7, 14], (iv) physiology [15, 16], (v) thermoregulation [17], (vi) evolution, ecology and diversity [18], (vii) chromosome evolution [19] and (viii) conservation strategies [2]. Nevertheless, the reproductive biology has not been studied, on physiological or molecular grounds, and little information on the transcriptome and gene expression related to physiological processes is available. The transcriptome analysis is an important tool to help identifying putative function of genes, translating the sequence of nucleotides in a sequence of amino acids, which is more likely to be conserved [20, 21]. In addition, the availability of a variety of bioinformatics tools allows the characterisation of these genes [22].

Some studies show that proteins with high expression levels in male reproductive tissues and with characteristics that meet the criteria of extracellular secretion, are good candidates to be considered as seminal fluid proteins (SFPs) [23–27]. The seminal fluid of insects contains sperm and a complex mixture of proteins, inorganic solutes, carbohydrates and lipids that are transferred to females during mating via the spermatophore. These proteins, which are produced in male gonads (testes, vas deferens and accessory glands), are important in the reproduction process by inducing physiological and behavioural changes in females, reducing responsiveness to other males, increasing the ovulation and egg laying rates, and altering feeding activity and also immune response [28–30].

The study of proteins transferred through the semen provides information for important biological questions such as the origin of new species and the origin of new molecules involved in sperm competition and coevolution between males and females [29, 31–36]. Seminal fluid proteins have two characteristics that according to several theoretical models might lead to speciation. i) These proteins are related with sexual selection and sexual conflict and ii) the rapid evolutionary rate of these proteins may also contribute to the evolution of reproductive barriers between populations. Furthermore, there is experimental evidences indicating a correlation between features that undergo the action of sexual selection and the speciation process [37–39].

In addition, several studies have demonstrated that many of these SFPs have similar characteristics to those found in the taxonomically restricted genes (TRGs), such as high evolutionary rate and low similarity between closely related species [25, 29, 40, 41]. According to Avila *et al*., the analysis of SFPs provides insight into the evolutionary patterns of reproductive traits [30]. Therefore, a better understanding of cicindelid reproductive molecules and their actions provides opportunities to reveal functionally conserved mechanism in cicindelids reproduction (highly conserved SFPs), as well as mechanisms involved in the reproductive isolation between species (lineage-specific SFPs) as a subset of seminal proteins is among the most rapidly evolving proteins [42–45].

To date, several SFPs have been described in insects, such as flies and mosquitoes (Diptera), field crickets (Orthoptera), honeybee (Hymenoptera), moths and butterflies (Lepidoptera) and in the beetle genus *Tribolium* [25, 26, 45–50]. Apart from the Polyphagan genus *Tribolium*, which is considered a model organism, no other species of beetles have been analysed for these proteins.

The aim of this study was to identify and characterise genes encoding proteins that are transferred to females during mating through seminal fluids in Cicindelids to contribute to the knowledge of the nature and function of insect SFPs and particularly in this ecologically important group of Adephagan beetles. To identify these proteins, EST libraries from gonads and accessory glands of male *Calomera littoralis* and *Cephalota litorea* were made and bioinformatically analysed. Proteins selected as candidate SFPs were submitted to gene expression analysis in female and male tissues. The complete sequence of genes showing differential expression patterns was obtained and the prediction of the function was inferred either by comparing with other insects or based on the sequence properties. Searches for homology of these putative SFP were performed in other cicidelid species. This work represents the first characterisation of putative SFP in Adephagan beetles.

## Results and discussion

### Library construction and EST assembly

Two separate cDNA libraries were constructed from RNA extracted from reproductive tissues (testes and accessory glands) of two males *C. littoralis* and one male of *C. litorea*. A total of 1,144 EST sequences were generated; 568 clones were sequenced from the *C. littoralis* library and 576 clones were sequenced from *C. litorea*. These sequences were trimmed for the removal of vector sequences and sequences <100 bp were excluded by Seqman (DNAstar, Inc. Madison, WI). EST sequences with high quality were previously deposited in GenBank under the following accession numbers: *C. litorea* (CV156657: CV157115) and *C. littoralis* (CV157116: CV157483). The high quality sequences of *C. littoralis* were assembled to 101 contigs (two or more sequences) and 84 singletons (single sequence), and *C. litorea* ESTs were assembled to 154 contigs and 58 singletons. Each cDNA library had a minimum average inset size ranging from 114 to 1,245 bp in *C. littoralis*, and from 139 to 1,246 in *C. litorea* library. The maximum number of ESTs that formed each contig was 15 ESTs in *C. littoralis* and 18 ESTs in *C. litorea* (Table 1).

**Table 1:** Summary of EST analyses from *C. littoralis* and *C. litorea* male gonad cDNA libraries

	*C. littoralis*	*C. litorea*
Number of ESTs generated	568	576
Number of High quality ESTs	368	459
Number of contigs	101	154
Number of singletons	84	58
Average length of contigs	523 bp	628 bp
Number of EST range in the contig	2-15	2-18

### Annotation – gene ontology

Blast2GO software showed that 82 contigs of *C. litorea* and 72 contigs of *C. littoralis* had no blast hits against the non-redundant protein database at National Center for Biotechnology Information (NCBI). The annotation of the 64 ESTs of *C. litorea* and 75 ESTs from *C. littoralis* were designated by database search algorithms BlastX for proteins in the NCBI web server (Table 2). Additionally, gene ontology annotations of all contigs were performed using Blast2GO.

**Table 2:** Summary of unigenes from *C. littoralis* and *C. litorea* male gonad analyses and annotated by Blast2go software

	*C. littoralis*	*C. litorea*
Annotated contigs	75	64
No annotated contigs	19	38
No mapping contigs	19	28
No blast hits contigs	72	82
No blast contigs	0	0
Total	185	212

**Fig. 1:**
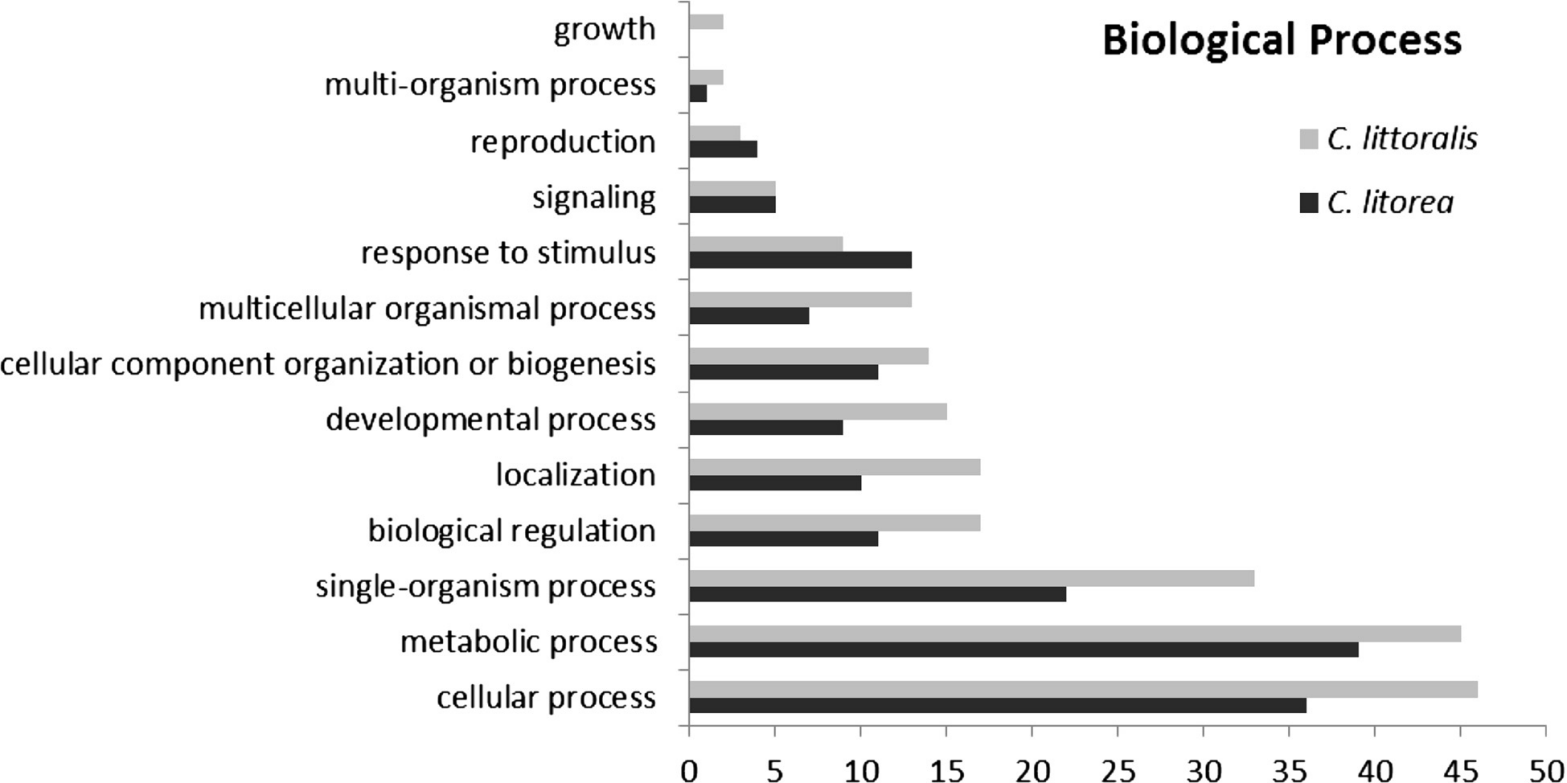
GO term distribution in biological processes for *C. littoralis* and *C. litorea* gonad unigenes. Percentages are in proportion to the total biological process GO annotations

**Fig. 2:**
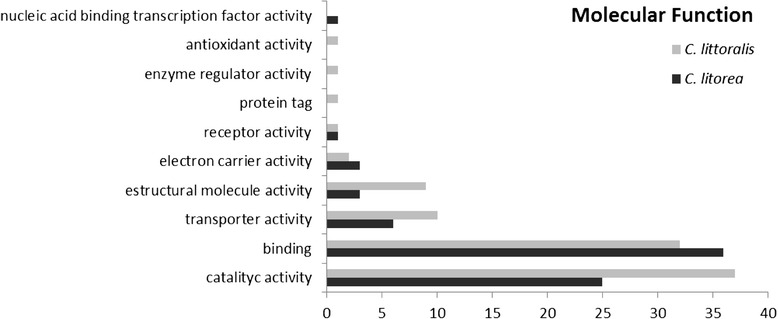
GO term distributions in the molecular functions for *C. littoralis* and *C. litorea* gonad unigenes. Percentages are in proportion to the total molecular function GO annotations

**Fig. 3:**
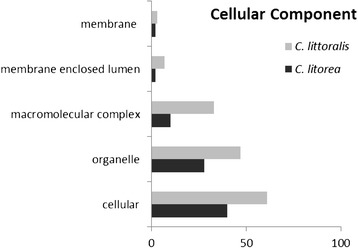
GO term distributions in the cellular components for *C. littoralis* and *C. litorea* gonad unigenes. Percentages are in proportion to the total cellular component GO annotations

Annotated sequences were classified according to their gene ontology (GO) into three categories: biological process, molecular function and cellular component (Fig. 1, 2 and 3). The proportions of genes associated with the different categories were highly similar among the two libraries; it is important to note that a sequence could be included in different categories and be associated to multiple GO. Within the category “Cellular Component”, the subcategories “cellular” and “organelle” were the most abundant in both libraries. However, the “extracellular” subcategory, where putative SFPs should be included, was not present in the analysis performed, and no sequences were annotated as SFPs. This could be due to both the low number of ESTs obtained in the cicindelid libraries and to the small number of Coleopteran libraries available for comparisons.

### Identification of putative SFPs

Nine genes putatively encoding SFPs were identified in *C. littoralis* and five in *C. litorea* by indirect approaches (Table 3). Candidate genes were selected after detection of one or more of the following characteristics i) the presence of a computationally predicted signal peptide inferred via Signal P 4.1 software [51, 52], ii) its localisation as extracellular and/or with plasma membrane destination inferred via ProtComp , iii) the recognition of a membrane helix inferred via TMHMM (InterproScan).

**Table 3:** Summary of the characteristics present in the candidate genes

	Contig	Cellular location	Signal peptide	Membrane helix
***C. littoralis***	13_59 (AcpC01)	Extracellular	No	Yes
	31_59	Plasma membrane	No	Yes
	50_59 (AcpC02)	Plasma membrane	No	Yes
	95_59	Extracellular	No	No
	129_59 (AcpC03)	Extracellular	Yes	Yes
	139_59	Plasma membrane	No	Yes
	161_59	Plasma Membrane	No	No
	171_59	Extracellular	No	No
	173_59	Plasma membrane	No	No
***C. litorea***	46_58	Plasma membrane	No	Yes
	70_58 (AcpC04)	Extracellular	No	No
	126_58	Plasma membrane	No	Yes
	101_58	Extracellular	Yes	No
	204_58	Extracellular	No	No

**Fig. 4:**
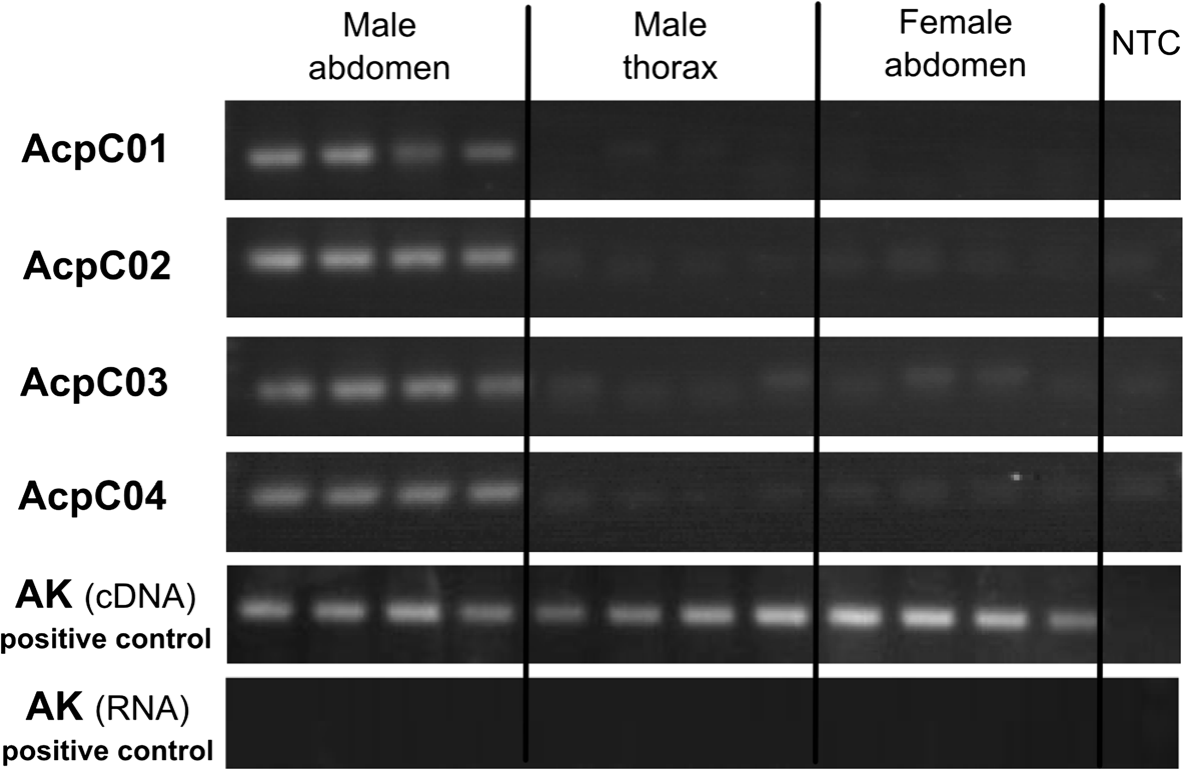
Results from RT-PCR, showing the expected pattern of expression in the different tissues for the four putative SFPs. Arginine kinase gene was used as positive control and was amplified from cDNA and RNA (free of DNAse) in all samples. NTC indicates negative template control

Of the 14 candidates, it was only possible to design useful RT-PCR primers for 12 (*C. littoralis*: eight and *C. litorea*: four). Tissue-specific expression patterns were obtained in the 12 available candidates. RT-PCR revealed strong amplification from the male abdomen but weak or not expression in female abdomens and male thoraxes in four of the genes (*C. littoralis*: AcpC01, AcpC02 and AcpC03 and *C. litorea*: AcpC04). The positive control gene (arginine kinase) amplified in all tissues (Fig. 4). The identification of an extracellular component and the tissue-specific patterns of expression found in these four genes suggest that they encode seminal fluid proteins. The other eight candidate genes with an identified extracellular component did not show any differential expression; some studies have demonstrated that not all SFPs have robust expression in male reproductive glands [29, 40], which could explain the low number of putative SFPs characterised in the present survey.

### SFP characterisation

Full length sequences and complete Open Reading Frames (ORFs) were obtained via Rapid amplification of cDNA ends PCR (RACE-PCR) for the four putative SFPs. Sizes ranged from 66 to 218 amino acids, which is in agreement with ACPs characterised in *Drosophila* [53] and references therein. Sequences are available in GenBank (KP164546–KP164549). Two of these proteins (AcpC01 and AcpC02) did not show any significant BlastX similarity (E-value > 10^−4^) against GenBank proteins, and appear to be novel proteins (Table 4). However, the protein AcpC01 yielded a similarity close to 30 % with two seminal fluid proteins (HACP050: *Heliconius hereto* and CSSFP014: *Chilo suppressalis*). This similarity, although low, may be a sign of the high evolutionary rates documented for some of these SFPs [43, 45, 54–56]. The proteins AcpC03 and AcpC04 showed similarity to sequences from *Drosophila yakuba* and *D. mojavensis,* respectively, which have not yet been characterised. Searches against several other insect genomes, run in Flybase, yielded the same results.

**Table 4:** Summary of the characterisation of cicindelids seminal fluid proteins

GEN	Amino acids size	CDS	GenBank hit	Flybase	PHYRE	Interpro	Protfun (gen ontology category)
**AcpC01**	66	Complete	No	No	NA	NA	Immune response
**AcpC02**	218	Complete	No	No	NA	NA	Growth factor
**AcpC03**	105	Complete	*Drosophila yakuba*/XP_002100905.1 (e-value 7e–4, 30 % similarity)	Yes/Dyak/GE17317-PA	NA	Svwc domine	Hormone
**AcpC04**	206	Complete	*Drosophila mojavensis*/XP_002007890.1 (e-value 8e–4, 30 % similarity)	Yes/Dmoj/GI12127-PA	NA	NA	Transcription regulation

The homologies and different functions assigned by the different software packages used are indicated

Additionally, we tried to determine protein structure (3D fold) using PHYRE protein fold recognition metaserver and protein domains using InterProScan software. PHYRE did not yield consistent results for any of the genes tested. This approach, which proves the annotation in the tertiary structure of the proteins, was useful to annotate *Drosophila* SFPs [44], suggesting that candidate cicindelid SFPs do not meet the criteria found in *Drosophila*, due to differences in the structure and/or function.

In the AcpC03 gene, a Single domain von Willebrand factor type C (SVWC) was detected via InterProScan. SVWC family proteins, which are largely present in arthropods, normally contain ten cysteines, and are thought to respond to environmental challenges, such as bacterial infection and nutritional status [57, 58]. Several studies have pointed out that SFPs may be involved in the immune response, as mating processes can transfer numerous pathogens into the female tract, jeopardising the reproductive success. Several SFPs analysed in *D. melanogaster* seem to have direct antimicrobial activity, protecting the male and subsequently the female reproductive tracts and even eggs against bacterial infection [59, 60] and/or stimulation of antimicrobial gene expression levels [61]. Other putative SFPs have been identified in other Diptera, such as *A. aegypti* and *A. gambiae* [48, 62], which are related to immune response. In Coleoptera, South *et al*. identified a putative SFP in *Tribolium* which is a predicted prophenoloxidase, an important component of the innate immune response in Arthropoda [50]. However, Protfun identifies AcpC03 as a hormone. This result could be in accordance with protein classes that are found in seminal proteins in different animals. Wolfner stated in her work that 40 % of accessory gland proteins appear to be peptide hormones or prohormones [63], and in *Drosophila melanogaster* ACP26Aa SFP was found to have similarity with califin C, a hormone from *Aplysia californica* [64] which is involved in the egg-laying process [65]. This could be an example of how a function assignment based on the sequence and structure similarity (InterproScan) could actually be different from a function assignment based not only on the structure but also on the physical/chemical and functional biological properties (Protfun). In other words, a conserved structure of a protein does not ensure a conserved function [66].

Finally, analysis with Protfun identified the gene AcpC01 to be an immune response protein. The other analyses did not assign a function to this protein based on similarity searches. However, Protfun analysis based on amino acid-derived input features did identify a function for this protein. This could be explained when considering the AcpC01gene as a novel putative SFP class that is either present only in tiger beetles (taxonomically restricted gene) or has not yet been characterised in other insects. The protein AcpC04 was identified as a transcription regulation factor by Protfun and also has similarities in Flybase and GenBank with a non-characterised protein in *D. mojavensis*. Although transcription regulation factors are not included within the described protein classes of SFP across animals [63], AcpC04 meets the requirements to be considered an SFP; therefore, further genetic studies may corroborate the biological function of this protein in tiger beetles. A similar consideration can be made in relation to the protein AcpC02, which according to the ontogenetic categories, is considered to be a growth factor by Protfun, although in this case, no homology has been found in the databases.

### Homology in cicindelid species

**Fig. 5:**
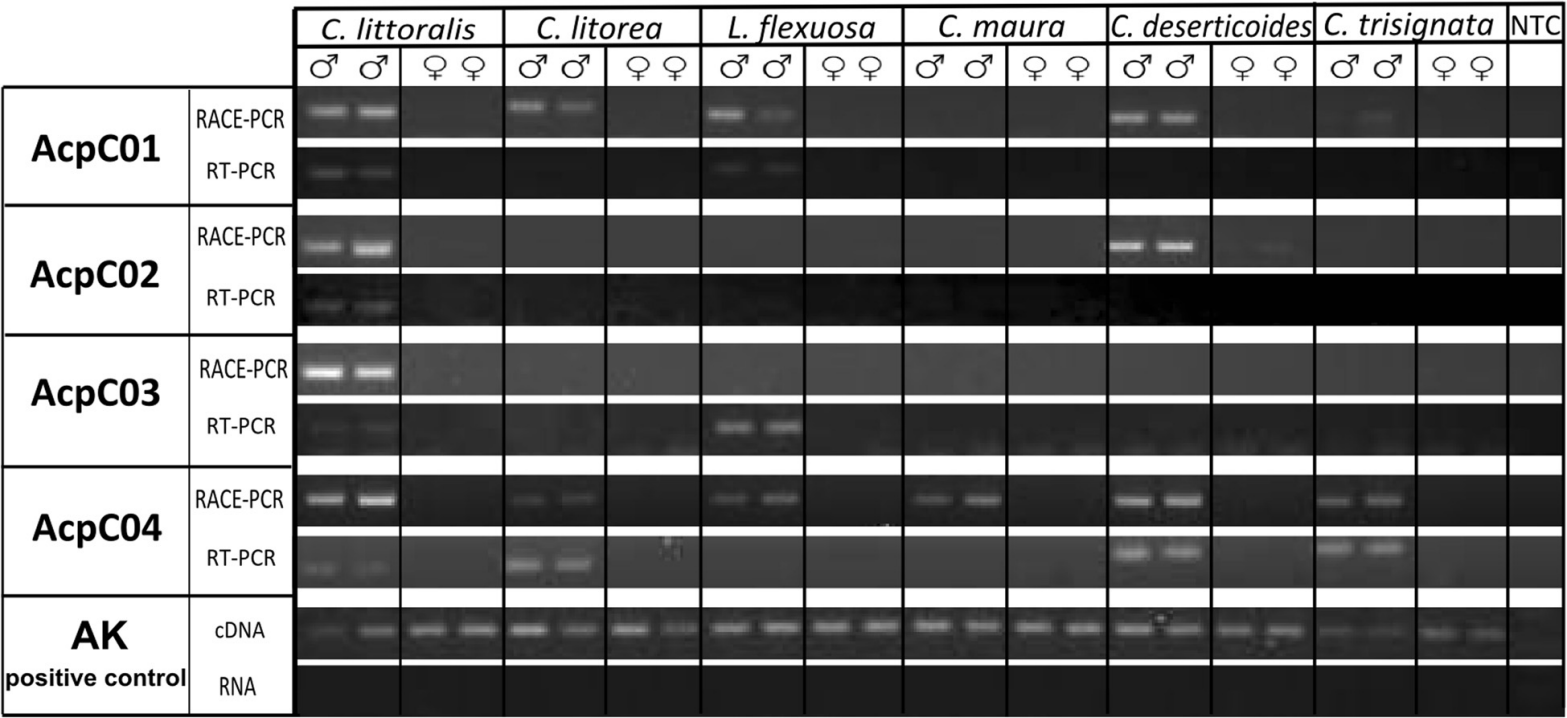
Results of the SFPs homology searches. RT-PCR and RACE-PCR primers were used to amplify the four putative SFPs in six cicindelid species. Arginine kinase gene was used as positive control and was amplified from cDNA and RNA (free of DNAse) in all samples. NTC indicates negative template control

**Fig. 6:**
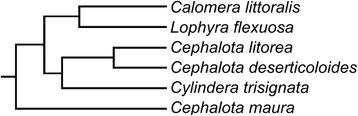
Phylogenetic tree based on COI sequences (unpublished data) from the cicindelids species analysed

RT-PCR and RACE-PCR primers were used in an attempt to amplify homologous sequences in the available cicindelid species (*C. litorea, C. littoralis, Lophyra flexuosa*, *Cephalota maura*, *Cephalota deserticoloides* and *Cylindera trisignata*). The AcpC04 gene, found originally in *C. litorea,* yielded homologous sequences in male abdomens of all of the analysed species (Fig. 5). This result is not surprising considering that although SFP are considered to have high evolutionary rate and low similarity between closely related species [25, 29, 40, 41], not all SFPs evolve rapidly and some loci are conserved between divergent taxa [25]. In this line of evidence, AcpC01 showed clear amplification in *C. litorea, C. littoralis, L. flexuosa* and *C. deserticoloides,* although weak signal in *C. trisignata* and no signal at all in *C. maura*. This result is coherent with the phylogenetic relationships obtained using cytochrome oxidase I gene (COI) (unpublished data) with the cicindelid species under study (Fig. 6). In fact *C. maura* is the most distantly related species.

However, AcpC03 showed amplification only in male abdomens of *L. flexuosa* (Fig. 5)*.* The phylogenetic tree (Fig. 6) showed that *C. littoralis* and *L. flexuosa* are closely related species. This close relationship could explain why these putative SFPs obtained from *C. littoralis* were also found in *L. flexuosa.* The lack of amplification in the other cicindelid species tested might be interpreted as a consequence of the rapid evolution that is generally considered for SFPs [42, 43, 45, 54–56, 67]. AcpC02 showed amplification in *C. littoralis* and *C. deserticoloides* with the RACE-PCR primers, but using the RT-PCR primers only *C. littoralis* gave positive results. However the detection of this gene in these two species suggest that it might be present in the most recent common ancestor of these two species, although with the primer pairs used was not detected.

## Conclusions

Two cDNA libraries were constructed from gonads of *C. littoralis* and *C. litorea* as a foundation to understanding the male reproductive system. A total of 568 and 576 ESTs were sequenced and analysed, assembled in 185 and 212 unigenes, respectively. Also, 75 and 64 contigs were annotated via Blast2go and no SFPs were found. We have identified 14 putative SFPs by bioinformatics analysis and found that four of them met the criteria of tissue-specific expression patterns, which led to them being considered as putative SFP. Functional annotation was difficult due to the fact that the four SFPs either do not show homology via similarity searches or reassemble with sequences whose function is unknown; only Interpro assigns a function in the immune response for AcpC03, as occurs with others insect SFPs. The predicted assignment of function via Protfun for AcpC01 and AcpC03 was immune response and hormone, respectively; these protein functions are included within the conserved protein classes of SFPs that have already been reported in several insect species. Searches for homology in other cicindelids showed that AcpC03 is only present in *C. littoralis* and *Lophyra flexuosa*, species which have a close phylogenetic relationship. SFPs are among the most rapidly evolving proteins and therefore this new putative SPF might represent a linage-specific SFP involved in reproductive isolation between species. In contrast, AcpC04 is present in all cicindelid species analysed here, and could be an example of a highly conserved SFP, at least in tiger beetles. The same can be said about AcpC01, with is present in all species tested except for the most distantly related species *C. maura*. This work represents the first identification of putative SFPs in tiger beetles that are one of the best studied worldwide distributed non-pest insects (more than 2,500 species described), are important in ecosystems as predators and are commonly used as bioindicators. The identification of cicindelid SFPs (both the rapidly evolving and the highly conserved) could represent a significant approach for understanding the male reproductive system in cicindelids and furthermore the species richness and adaptive evolution in Adephagan beetles.

## Methods

### Library construction and EST assembly

#### Tiger beetles

Two cDNA libraries were constructed from male reproductive tissue (testes and accessory glands) of two *Calomera littoralis* males and a *Cephalota litorea* male. The developmental stage of the testes was that of sexually mature males with the final half of the testes full of spermatozoa (pearl white colour) and the anterior part with active meiosis (transparent white colour). Reproductive tissues were extracted and preserved in RNAlater (Qiagen, Crawley, UK) and stored at −20 °C prior to extraction.

#### cDNA libraries

According to the manufacturer’s instructions, total RNA was precipitated using the RNeasy Protect Mini Kit (Qiagen, Crawley, UK). The RNA sample concentrations were quantified by spectrometry. mRNA was purified using the Oligotex ARNm mini kit (Qiagen, Crawley, UK). The BD SMART PCR cDNA Synthesis kit (BD Biosciences) was used for cDNA libraries construction. The first strand was synthesised using BD PowerScript Reverse Transcriptase, the SMART IIA Oligonucleotide and the CDS IIA primer provided in the kit. The double-stranded cDNA was synthesised by PCR and purified using Micropure-EZ (Millipore). The cDNA products were analysed using agarose gel electrophoresis to determine their quality before cloning. cDNA was ligated into a TOPO vector and was transformed by TOPO TA cloning (Invitrogen) using TOP10 chemically competent *E. coli* cells. Libraries were plated on LB medium and grown at 37 °C overnight. Colonies were manually picked up for PCR amplification with M13 and T7 universal vector primers and subsequent sequencing with poly-T primer on an ABI Prism 3,700 sequencer (Applied Biosystems).

### Annotation – gene ontology

The Seqman module of DNAStar (ver. Madison, WI) was used to remove the vector sequence, trim ends using program defaults and assemble sequences. Assembling parameters were: 80 % for minimal match percentage, 100 for minimal sequence length, 0 for gap penalty, and 0. 7 for gap length penalty.

Assembled sequences (contigs and singletons) were subjected to a similarity search for assigned putative protein functions using BlastX of the Blast2GO v2.5.0 software [68] with 10^−3^ for the cut-off E-value. BlastX reference was used from the non-redundant protein database of GenBank in the NCBI.

Gene ontology enrichment analysis was performed with Blast2GO mapping to determine protein functions in biological processes.

### Identification of putative SFP

Indirect strategies based on bioinformatics tools were previously employed in insects to identify putative SFP unigenes [25, 27, 40, 69]. First, ORF of each unigene generated in Orfinder (http://www.ncbi.nlm.nih.gov/gorf/gorf.html) were selected and SignalP 4.1 [70] software was used to identify a predicted signal peptide. Additionally, integral prediction of protein location was analysed by ProtComp v9 software (http://www.softberry.com) and TMHMM tool in InterProScan [71] was used to determine the presence of a membrane helix. Those candidate genes with similar predicted functions to known SFPs (extracellular and/or with membrane destination, as the signal peptide can sometimes be recognised as a membrane helix) were also selected.

Second, patterns of tissue-specific expression were examined for each candidate SFP via RT-PCR. PCR primers were designed with PrimerExpress 3.0 (Applied Biosystems) (Table 5). Total RNA was isolated from the male abdomen, male thorax (pronotum) and female abdomen, in three males and three females of *C. littoralis* and *C. litorea*. RNA was extracted using the RNeasy Protect Mini Kit (Qiagen) following the manufacturer’s protocol. Around 1,2 μg of each RNA extraction were treated with TURBO DNA free (Ambion, Life Technologies) and reverse-transcribed using the QuantiTect Reverse Transcription kit (Qiagen). One μl of a ten-fold dilution of cDNA was used as template for a 12,5 μl RT-PCR experiments. PCR was performed using the following cycling parameters: one cycle of 2 min at 96 °C, 35 cycles of 30 s at 96 °C, 30 s at 60 °C and 1 min at 72 °C, and a final extension of 10 min at 72 °C. The arginine kinase gene (AK) was used as a positive control using in this case cDNA and also RNA (DNAse treated) to discard genomic DNA amplification. PCR amplicons were electrophoresed on agarose gel with RedSafe™ (INTRON Biotechnologies, Korea).

**Table 5:** RACE-PCR and RT-PRC primers for the four putative seminal fluid proteins and the amplicon sizes

	Primers sequence	Amplicon size
**AcpC01**	**RACE_F1** 5′-GTATTCCATTGTGTCCACCACCTCCGG-3′	**128**
	**RACE_R1** 5′-TGGTGGACAAGGTGGACAACATGGAAC -3′	
	**RT_F1** 5′-TTGCCCTCCATGTGCAGTAC-3′	**139**
	**RT_R1** 5′-TGGCTTCTGTGGCTCAAATTT-3′	
**AcpC02**	**RACE_F2** 5′- TGAGGAACCAGCCGCACAAGTAAAGAC-3′	**191**
	**RACE_R2** 5′-AGACCGACTCTGCAGTTTTTGTCTCGG-3′	
	**RT_F2** 5′-AGGAACCAGCCGCACAAGTA-3′	**110**
	**RT_R2** 5′-CTCCTTGTTGGGTGGTGCAT-3′	
**AcpC03**	**RACE_F3** 5′-TCATAACGATGATTCTGCCGCTCGTGG-3′	**176**
	**RACE_R3** 5′-GACACTCGAGATGCCTACAGTCCGGTA-3′	
	**RT_F3** 5′-ATGCTGTGCTGCTTGTGCAT-3′	**100**
	**RT_R3** 5′-GGACAACAGGCCGGAAATG-3′	
**AcpC04**	**RACE_F4** 5′-ACCAGTTTGTGATTGTCCGCCGTTACG-3′	**300**
	**RACE_R4** 5′-GTGTAACTGAACGCACGGGAAATAGCC-3′	
	**RT_F4** 5′-GCTATTTCCCGTGCGTTCAG-3′	**100**
	**RT_R4** 5′-CGGAGATCTCGTCTGCGTTT-3′	

### SFP characterisation

The SMARTer™ RACE cDNA Amplification Kit (Clontech Laboratories, Inc. Kyoto, Japan) was used to obtain the full length cDNA sequences. Total RNA from one *C. littoralis* male and one *C. litorea* male was used to obtain the first strand of 3′ and 5′ RACE Ready CDNA. Gene-specific primers for 5′ and 3′ RACE were designed (Table 5). The synthesis of first strand cDNA was performed following PCR conditions, as indicated the manufacturer’s instructions. The amplification products were sequenced in triplicate by SAI at the University of Murcia (Spain) using an ABI Prism 3,130 Sequencer (Applied Biosystems) and assembled by GENEIOUS v5 [72] to obtain the complete cDNA sequence.

The complete ORFs derivate from the full length sequences were generated in Orfinder (http://www.ncbi.nlm.nih.gov/gorf/gorf.html) and were used as queries in BlastX to search for homologues in other species with an e-value cut-off of 10^−4^ and identities >30 %. We also searched for similarity between our putative SFPs and other known insect proteins using Flybase [73]. Additionally, protein domains were searched again using InterProScan [74] and sequences were submitted to the PHYRE protein fold recognition server [75] to generate the protein structure.

Sequences were submitted to ProtFun 2.2 Server (http://www.cbs.dtu.dk/services/ProtFun/) for analysis of the GO ontology to predict function based on amino acid sequence-derived input features (physical/chemical and functional biological properties) such as predicted protein secondary structure, transmembrane helices, subcellular localisation and post-transcriptional modifications [66].

### Homology in cicindelid species

To determine the presence/absence of these putative SFPs in other species, total RNA was extracted from the abdomen of two males and the abdomen of two females of the following species: *Lophyra flexuosa*, *Cephalota maura*, *Cephalota deserticoloides* and *Cylindera trisignata.* RNA was extracted using the RNeasy Protect Mini Kit (Qiagen) following the manufacturer’s instructions. For each tissue type around 1, 2 μg of Extracted RNA was treated with TURBO DNA free (Ambion, Life Technologies) to remove DNA contamination, and reverse-transcribed using the QuantiTect Reverse Transcription kit (Qiagen). RACE-PCR and RT-PCR primers that had been previously designed (Table 5) were tested in all of the available samples under the same conditions previously described for each PCR reaction. The arginine kinase gene (AK) was used as a positive control using cDNA and RNA.

## Abbreviations

EST: Expressed sequence tag
GO: Gene ontology
ORF: Open reading frame
RACE: Rapid amplification of cDNA ends polymerase chain reaction
RT-PCR: Reverse transcription polymerase chain reaction
SFP: Seminal fluid protein
TRG: Taxonomically restricted genes

## References

[1] Pearson DL (1992). Tiger beetles as indicators for biodiversity patterns in Amazonia. Res Explor.

[2] Pearson DL, Cassola F (1992). World-wide species richness patterns of tiger beetles (Coleoptera: Cicindelidae): indicator taxon for biodiversity and conservation studies. Conserv Biol.

[3] Carroll SS, Pearson DL (1998). Spatial modeling of butterfly species richness using tiger beetles (Cicindelidae) as a bioindicator taxon. Ecol Appl.

[4] Rodriguez JP, Pearson DL, Barrera R (1998). A test for the adequacy of bioindicator taxa: are tiger beetles (Coleoptera: Cicindelidae) appropriate indicators for monitoring the degradation of tropical forests in Venezuela?. Biol Conserv.

[5] Cassola F, Pearson DL (2000). Global patterns of tiger beetle species richness (Coleoptera: Cicindelidae): their use in conservation planning. Biol Conserv.

[6] Pearson DL, Cassola F (2005). A quantitative analysis of species descriptions of tiger beetles (Coleoptera: Cicindelidae), from 1758 to 2004, and notes about related developments in biodiversity studies. Coleops Bull.

[7] Pearson DL (1988). Biology of Tiger Beetles. Ann Rev Entomol.

[8] Pearson DL, Blum MS, Jones TH, Fales HM, Gonda E, White BR (1988). Historical perspective and the interpretation of ecological patterns: defensive compounds of tiger beetles (Coleoptera: Cicindelidae). Am Nat.

[9] Willis HL (1967). Bionomics and zoogeography of tiger beetles of saline habitats in the central United States (Coleoptera: Cicindelidae). Univ Kans Sci Bull.

[10] Singh T, Gupta S (1982). Morphology and histology of the mandibular gland in Cicindela sexpunctata Fabr. ( Coleoptera: Cicindeliadae). Uttar pradesh. J Zool.

[11] Horn W, Wystman P (1915). Coleoptera Adephaga (family Carabidae, subfamily Cicindelinae). Genera insectorum.

[12] Horn W. Pars 86: Carabidae: Cicindelinae. In Junk and Schenkling Berlin editors. Belin: W.Junk; *Coleopterum catalogus*. 1926.

[13] Wiesner J, Verzerichnis der Sabdlaufkäfer der Welt (1992). Checklist of the tiger beetles of the world.

[14] Sota T, Danks HV (1994). Variation of carabid life cycles along climatic gradients: an adaptive perspective for life-history evolution under adverse conditions. Insect life-cycle polymorphism: theory, evolution; and ecological consequences for seasonal and diapause control.

[15] Spangler HG (1988). Hearing in tiger beetles (Cicindelidae). Physiol Entomol.

[16] Toh Y, Okamura JY (2001). Behavioural responses of the tiger beetle larva to moving objects: role of binocular and monocular vision. J Exp Biol.

[17] Dreisig H (1980). Daily activity, thermoregulation and water loss in the tiger beetle *Cicindela hybrid*. Oecologia.

[18] Pearson DL, Vogler AP (2001). Tiger beetles: the evolution, ecology and diversity of the cicindelids.

[19] Galián J, Proença SJR, Vogler AP (2007). Evolutionary dinamics of autosomal-heterosomal rearrangements in a multiple-X chromosome system of tiger beetles (Cicindelidae). BMC Evol Biol.

[20] Haas BJ, Volfovsky N, Town CD, Troukhan M, Alexandrov N, Feldmann KA (2002). Full-length messenger RNA sequences greatly improve genome annotation. Genome Biol.

[21] Nagaraj SH, Gasser RB, Ranganathan S (2007). A hitchhiker’s guide to expressed sequence tag (EST) analysis. Brief Bioinform.

[22] Ayroles JF, Carbone MA, Stone EA, Jordan KW, Lyman RF, Magwire MM (2009). Systems genetics of complex traits in *Drosophila melanogaster*. Nat Genet.

[23] Davies SJ, Chapman T (2006). Identification of genes expressed in the accessory glands of male Mediterranean fruit flies (*Ceratitis capitata*). Insect Biochem Mol Biol.

[24] Walters JR, Harrison RG (2008). EST analysis of male accessory glands from *Heliconius* butterflies with divergent mating systems. BMC Genomics.

[25] Walters JR, Harrison RG (2010). Combined EST and proteomic analysis identifies rapidly evolving seminal fluid proteins in *Heliconius* butterflies. Mol Biol Evol.

[26] Almeida FC, DeSalle R (2009). Orthology, function and evolution of accessory gland proteins in the Drosophila repleta group. Genetics.

[27] Scolari F, Gomulski LM, Ribeiro JMC, Siciliano P, Meraldi A, Falchetto M (2012). Transcriptional profiles of mating-responsive genes from testes and male accessory glands of the Mediterranean fruit fly. Ceratitis capitata PLoS ONE.

[28] Haerty W, Jagadeeshan S, Kulathinal RJ, Wong A, Ravi Ram K, Sirot LK (2007). Evolution in the fast lane: rapidly evolving sex-related genes in drosophila. Genetics.

[29] Findlay GD, Yi XH, MacCoss MJ, Swanson WJ (2008). Proteomics reveals novel *Drosophila* seminal fluid proteins transferred at mating. PLoS Biol.

[30] Avila FW, Sirot LK, LaFlamme BA, Rubinstein CD, Wolfner MF (2011). Insect seminal fluid proteins: identification and function. Annu Rev Entomol.

[31] McGraw LA, Gibson G, Clark AG, Wolfner MF (2004). Genes regulated by mating, sperm, or seminal proteins in mated female *Drosophila melanogaster*. Curr Biol.

[32] Fiumera AC, Dumont BL, Clark AG (2005). Sperm competitive ability in *Drosophila melanogaster* associated with variation in male reproductive proteins. Genetics.

[33] Wigby S, Chapman T (2005). Sex peptide causes mating costs in female *Drosophila melanogaster*. Curr Biol.

[34] Wigby S, Sorot LK, Linkalater JR, Buehner N, Calboli FCF, Bretman A (2009). Seminal fluid protein allocation and male reproductive success. Curr Biol.

[35] Fedorka KM, Winterhalter WE, Ware B (2011). Perceived sperm competition intensity influences seminal fluid protein production prior to courtship and mating. Evolution.

[36] LaFlamme BA, Ram KR, Wolfner MF (2012). The *Drosophila melanogaster* seminal fluid protease “seminase” regulates proteolytic and post-mating reproductive processes. PLoS Genet.

[37] Ritchie MG (2007). Sexual selection and speciation. Annu Rev Ecol Evol Syst.

[38] Snook RR, Chapman T, Moore PJ, Wedell N, Crudgington HS (2009). Interactions between the sexes: new perspectives on sexual selection and reproductive isolation. Evol Ecol.

[39] Castillo DM, Moyle LC (2014). Intraspecific sperm competition genes enforce post-mating species barriers in Drosophila. P Roy Soc B-Biol Sci.

[40] Findlay GD, MacCoss MJ, Swanson WJ (2009). Proteomic discovery of previously unannotated, rapidly evolving seminal fluid genes in *Drosophila*. Genome Res.

[41] Marshall JL, Huestis DL, Garcia C, Hiromasa Y, Wheeler S, Noh S (2011). Comparative proteomics uncovers the signature of natural selection acting on the ejaculate proteomes of two cricket species isolated by post-mating pre-zygotic phenotypes. Mol Biol Evol.

[42] Clark NL, Aagaard JE, Swanson WJ (2006). Evolution of reproductive proteins from animals and plants. Reproduction.

[43] Clark NL, Swanson WJ (2005). Pervasive adaptive evolution in primate seminal proteins. PLoS Genet.

[44] Mueller JL, Ripoll DR, Aquadro CF, Wolfner MF (2004). Comparative structural modelling and inference of conserved protein classes in *Drosophila* seminal fluid. Proc Natl Acad Sci U S A.

[45] Andres JA, Maroja LS, Bogdanowicz SM, Swanson WJ, Harrison RG (2006). Molecular evolution of seminal proteins in field crickets. Mol Biol Evol.

[46] Andres JA, Maroja LS, Harrison RG (2008). Searching for candidate speciation genes using a proteomic approach: seminal proteins in field crickets. Proc R Soc Lond B Biol Sci.

[47] Collins AM, Caperna TJ, Williams V, Garrett WM, Evans JD (2006). Proteomic analyses of male contributions to honey bee sperm storage and mating. Insect Mol Biol.

[48] Sirot LK, Poulson RL, McKenna MC, Girnary H, Wolfner MF, Harrington LC (2008). Identity and transfer of male reproductive gland proteins of the dengue vector mosquito, *Aedes aegypti*: potential tools for control of female feeding and reproduction. Insect Biochem Mol Biol.

[49] Baer B, Heazlewood JL, Taylor NL, Eubel H, Millar AH (2009). The seminal fluid proteome of the honeybee *Apis mellifera*. Proteomics.

[50] South A, Sirot LK, Lewis SM (2011). Identification of predicted seminal fluid proteins in *Tribolium castaneum*. Insect Mol Biol.

[51] Nielsen H, Engelbrecht J, Brunak S, von Heijne G (1997). Identification of prokaryotic and eukaryotic signal peptides and prediction of their cleavage sites. Protein Eng.

[52] Bendtsen JD, Nielsen H, von Heijne G, Brunak S (2004). Improved prediction of signal peptides: signalP 3.0. J Mol Biol.

[53] Chapman T, Davies SJ (2004). Functions and analysis of the seminal fluid proteins of male Drosophila *melanogaster* fruit flies. Peptides.

[54] Swanson WJ, Clark AG, Waldrip-Dail HM, Wolfner MF, Aquadro CF (2001). Evolutionary EST analysis identifies rapidly evolving male reproductive proteins in *Drosophila*. Proc Natl Acad Sci U S A.

[55] Swanson WJ, Vacquier VD (2002). The rapid evolution of reproductive proteins. Nat Rev Genet.

[56] Clark AG, Aguade M, Prout T, Harshman LG, Langley CH (1995). Variation in sperm displacement and its association with accessory-gland protein loci in *Drosophila melanogaster*. Genetics.

[57] Sheldon TJ, Miguel-Aliaga I, Gould AP, Taylor WR, Conklin D (2007). A novel family of single VWC-domain proteins in invertebrates. FEBS Lett.

[58] Zinke I, Schutz CS, Katzenberger JD, Bauer M, Pankratz MJ (2002). Nutrient control of gene expression in *Drosophila*: microarray analysis of starvation and sugar-dependent response. EMBO J.

[59] Samakovlis C, Kylsten P, Kimbrell DA, Engstrom A, Hultmark A (1991). The andropin gene and its product, a male-specific antibacterial peptide in *Drosophila melanogaster*. EMBO J.

[60] Lung O, Kuo L, Wofner MF (2001). Drosophila males transfer antibacterial proteins from their accessory gland and ejaculatory duct to their mates. J Insect Physiol.

[61] Mueller JL, Page JL, Wolfner M (2007). An ectopic expression screen reveals the protective and toxic effects of *Drosophila* seminal fluid proteins. Genetics.

[62] Rogers DW, Whitten MMA, Thailayil J, Soichot J, Levashina EA, Catteruccia F (2008). Molecular and cellular components of the mating machinery in *Anopheles gambiae* females. Proc Natl Acad Sci U S A.

[63] Wolfner MF (2009). Battle and Ballet: molecular interactions between the sexes in *Drosophila*. J Hered.

[64] Heifetz Y, Oliver L, Frongillo EA, Wolfner MF (2000). The Drosophila seminal fluid protein Acp26Aa stimulates release of oocytes by the ovary. Curr Biol.

[65] Rothman BS, Hawke DH, Brown RO, Lee TD, Dehghan AA, Shively JE (1986). Isolation and primary structure of the califins, three biologically active egg-laying hormone-like peptides from the atrial gland of *Aplysia californica*. J Biol Chem.

[66] Jensen LJ, Ussery DW, Brunak S (2003). Functionality of system components: conservation of protein function in protein feature space. Genome Res.

[67] Panhuis TM, Clark NL, Swanson WJ (2006). Rapid evolution of reproductive proteins in abalone and *Drosophila*. Philos Trans R Soc B.

[68] Conesa A, Gotz S, Garcia-Gomez J, Terol J, Talon M, Robles M (2005). Blast2GO: a universal tool for annotation, visualization and analysis in functional genomics research. Bioinformatics.

[69] Sonenshine DE, Bissinger BW, Egekwu N, Donohue KV, Khalil SM (2011). First transcriptome of the testis-vas deferens-male accessory gland and proteome of the spermatophore from *Dermacentor variabilis* (Acari: Ixodidae). PLoS ONE.

[70] Nordahl Petersen T, Brunak S, von Heijne G, Nielsen H (2011). SignalP 4.0: discriminating signal peptides from transmembrane regions. Nat Methods.

[71] Zdobnov EM, Apweiler R (2001). InterProScan–an integration platform for the signature-recognition methods in InterPro. Bioinformatics.

[72] Drummond AJ, Ashton B, Buxton S, Cheung M, Cooper A, Duran C et al*.* Geneious v5.5 [http://www.geneious.com] 2010

[73] St Pierre SE, Ponting L, Stefancsik R, McQuilton P, the FlyBaseConsortium (2014). FlyBase 102-advanced approaches to interrogating FlyBase. Nucleic Acids Res.

[74] Jones P, Binns D, Chang HY, Fraser M, Li W, McAnulla C (2014). InterProScan 5: genome-scale protein function classification. Bioinformatics.

[75] Bennett-Lovsey RM, Herbert AD, Sternberg MJE, Kelley LA (2008). Exploring the extremes of sequence/structure space with ensemble fold recognition in the program Phyre. Proteins.

